# Molecular characteristics of synchronous multiple gastric cancer

**DOI:** 10.7150/thno.42814

**Published:** 2020-04-07

**Authors:** Anqiang Wang, Zhongwu Li, Meng Wang, Shuqin Jia, Jiahu Chen, Ke Ji, Xin Ji, Xianglong Zong, Xiaojiang Wu, Ji Zhang, Ziyu Li, Lianhai Zhang, Ying Hu, Zhaode Bu, Qi Zheng, Jiafu Ji

**Affiliations:** 1 Department of Gastrointestinal Surgery, Key Laboratory of Carcinogenesis and Translational Research (Ministry of Education), Peking University Cancer Hospital & Institute, Beijing 100142, China; 2 Department of Pathology, Key Laboratory of Carcinogenesis and Translational Research (Ministry of Education), Peking University Cancer Hospital & Institute, Beijing 100142, China; 3 Novogene Bioinformatics Technology Co., Ltd, Beijing 100083, China; 4 Center for Molecular Diagnostics, Key laboratory of Carcinogenesis and Translational Research (Ministry of Education), Peking University Cancer Hospital & Institute, Beijing 100142, China; 5 Department of Biobank, Key laboratory of Carcinogenesis and Translational Research (Ministry of Education/Beijing), Peking University Cancer Hospital & Institute, Beijing 100142, China

**Keywords:** Multiple gastric cancer, whole-exome sequencing, clonal relationship, *MSH2* gene, predisposing gene, TCGA

## Abstract

**Rationale**: Multiple gastric cancer (MGC) is characterized by the presence of more than two different tumors in the stomach. However, the clonal relationship and carcinogenesis of MGC remain unclear. We investigated the clonal relationship and role of germline mutations in the carcinogenesis of MGC.

**Methods:** We gathered 16 multiple gastric cancer patients. Thirty-three tumor samples and sixteen normal gastric tissue or blood samples were obtained from January 2016 to December 2017. We also conducted analyses for 208 gastric cancer and 49 esophagogastric junction cancer (GC-EGJ) tumors from TCGA. DNA extraction from our samples was conducted for whole-exome sequencing (WES).

**Results**: Tumor mutation burden (TMB) was not statistically significant within database and our data in the GC-EGJ (P=0.0591) and GC groups (P=0.3113). The mutation spectrum and signatures also showed uniform distributions in GC and GC-EGJ groups within our data and TCGA database. Among sixteen patients, four were identified as monoclonal, in which 11, 10, 26 and 6 somatic mutations were shared within different tumors of P7, P8, P9 and P16, respectively. However, no common mutation between different tumors of the same patient was found among the other 12 patients. After identifying predisposing genes, we found that germline *MSH2* and *NCOR2* mutations were significantly dominant in 8/12 and 10/12 of genetic MGC patients. Additionally, all patients were identified with *MSH2* mutations in cancer samples of those genetic MGC patients. Taking genetic MGCs as a whole, we identified that *TP53* were significantly mutated in 14 of 25 tumor samples.

**Main conclusions**: WES analyses are suggestive of monoclonal and polyclonal origin of MGC, which may promote the classification of MGC into genetic and metastatic MGC. For patients with genetic MGC, germline *MSH2 X314*_splice variants may contribute to carcinogenesis, thus prompting the consideration of more radical surgery and/or anti-PD-1/PD-L1 therapy.

## Introduction

Gastric cancer is one of the most common gastrointestinal malignant cancers, with approximately one million of new patients diagnosed and 782.7 thousand patients succumbing to the disease every year 1. The incidence of gastric cancer ranks sixth among all types of cancer, and it has the second highest cancer-related death rate 1, 2. Multiple gastric cancer (MGC) refers to a special type of gastric cancer present in more than two different sites of the stomach, with reported rates ranging from 6-14% 3-5.

As one type of rare cancer, multiple gastric cancer is rigorously defined. Firstly, the different focal sites must be confirmed as cancer. Second, the different cancer tissues should be separated by normal gastric mucosa. Finally, the various tumors must exclude metastasis from each other^5^. However, the different tumors within the normal tissue may not be totally equal to independent cancer, which should be identified using molecular biological techniques. A remaining question is whether the widely defined MGC is monoclonal or multicentric in origin.

Compared with solitary gastric cancer, multiple gastric cancer is more prevalent among elderly male patients and in the upper stomach 6-8. However, the differences in vascular cancer embolus, differentiation state, lymph node metastasis and other clinical pathological characteristics between solitary and multiple gastric cancers are not statistically significant. Endoscopic tumor dissection, subtotal gastrectomy and total gastrectomy are potential treatments for different patients with various stage tumors. According to survival analyses, the differences in survival between patients with solitary and multiple gastric cancers are not statistically significant 8-10. However, most analyses were confined to the early stage of tumors. Researchers have not clearly determined whether a difference in survival exists between patients with multiple gastric cancer of different origins.

Other challenges include whether patients with MGCs have definite predisposing genes and surgical methods for patients with obvious familial aggregation who are carrying susceptibility genes. The E-cadherin (*CDH1*) gene, encoding the E-cadherin protein, is a cancer predisposition gene predominantly mutated in hereditary diffuse gastric cancer (HDGC). The cumulative incidence of gastric cancer could reach 70% and 52% for male and female patients with positive *CDH1* mutations, respectively 11. In addition to *CDH1* mutations, mutations in many other genes involved in homologous recombination such as *BRCA1, BRCA2, PALB2* and others, were also reported to increase the risk of gastric cancer 12-14. Whether the occurrence of MGCs in patients could also attribute to some predisposing genes deserve research. Meanwhile, Huntsman 15 conducted total gastrectomy in young persons with truncating mutations in *CDH1* from two unrelated families with hereditary diffuse gastric cancer. Multiple gastric cancer was observed in 60% (3/5) of young persons, and the young asymptomatic carriers of germ-line truncating *CDH1* mutations were advised to receive genetic counselling and consider prophylactic gastrectomy for highly penetrant hereditary diffuse gastric cancer. Therefore, a clear answer is not available for the question of whether a part of multiple gastric cancer also belongs to hereditary gastric cancer and how to perform the correct operation for these patients.

Although the clinical and pathological characteristics, treatment modalities and overall survival rates are not significantly different between patients with solitary and multiple gastric cancers, the reliability of the research evidence should be questioned due to the use of different definitions and classifications. We performed whole-exome sequencing of different tumour samples from patients with multiple gastric cancer to validate the clonal relationships of different tumours, improve the classification and study the carcinogenesis of MGC.

## Methods

### Patients and tissue samples

We recruited 16 patients with multiple gastric cancer who underwent subtotal and total gastrectomy at Peking Cancer Hospital from January 2016 to December 2017. Thirty- three tumor samples and sixteen normal gastric tissue or blood samples were obtained for the experiment. Detailed clinical information was collected from every patient, and the pathological diagnosis of every tumor tissue was confirmed again by two independent pathologists. We performed HE staining for sections from every tumor resected from patients with multiple gastric cancer. Informed consent was obtained from every patient. The study protocol was approved by the ethical committee of Peking Cancer Hospital.

### DNA collection and whole-exome sequencing

We performed HE staining for each tumor sample and collected cancer cells from 5-μm thick formalin-fixed paraffin-embedded (FFPE) tissue sections. DNA was extracted from thirty-three tumor samples for the experiments and sixteen blood samples as controls. Whole-exome sequencing was conducted on Illumina HiSeq 4000.

### Sequencing data analysis

WES data were analyzed for somatic mutations, insertions and deletions (INDEL), copy number variants (CNVs), the mutation spectrum, mutation signatures, significant mutated genes (SMGs), driver genes and predisposing genes. The purity and ploidy of tumor samples were analyzed using ABSOLUTE software 16. A phylogenetic tree was constructed from the somatic mutations detected in each tumor sample to explore the clonal relationships of different tumors from every patient with multiple gastric cancer. A PyClone analysis 17 was also performed to further validate the clonal relationships of each patient. CNVs detected in different tumour samples from the same patients were also analysed and compared to observe the inherent links as supplementary evidence. The significant mutated genes and driver mutations were analyzed for tumor samples.

### Public database mining

We extracted 208 gastric adenocarcinoma (GC) samples and 49 esophagogastric junction adenocarcinoma (GC-EGJ) samples from the cancer genome atlas (TCGA) database 18. We compared the tumor mutation burden (TMB), mutation spectrum and mutation signatures among our sequencing data and the publicly available data.

## Results

### The distributions of clinical-pathological characteristics among patients with multiple gastric cancer

**Figure 1:**
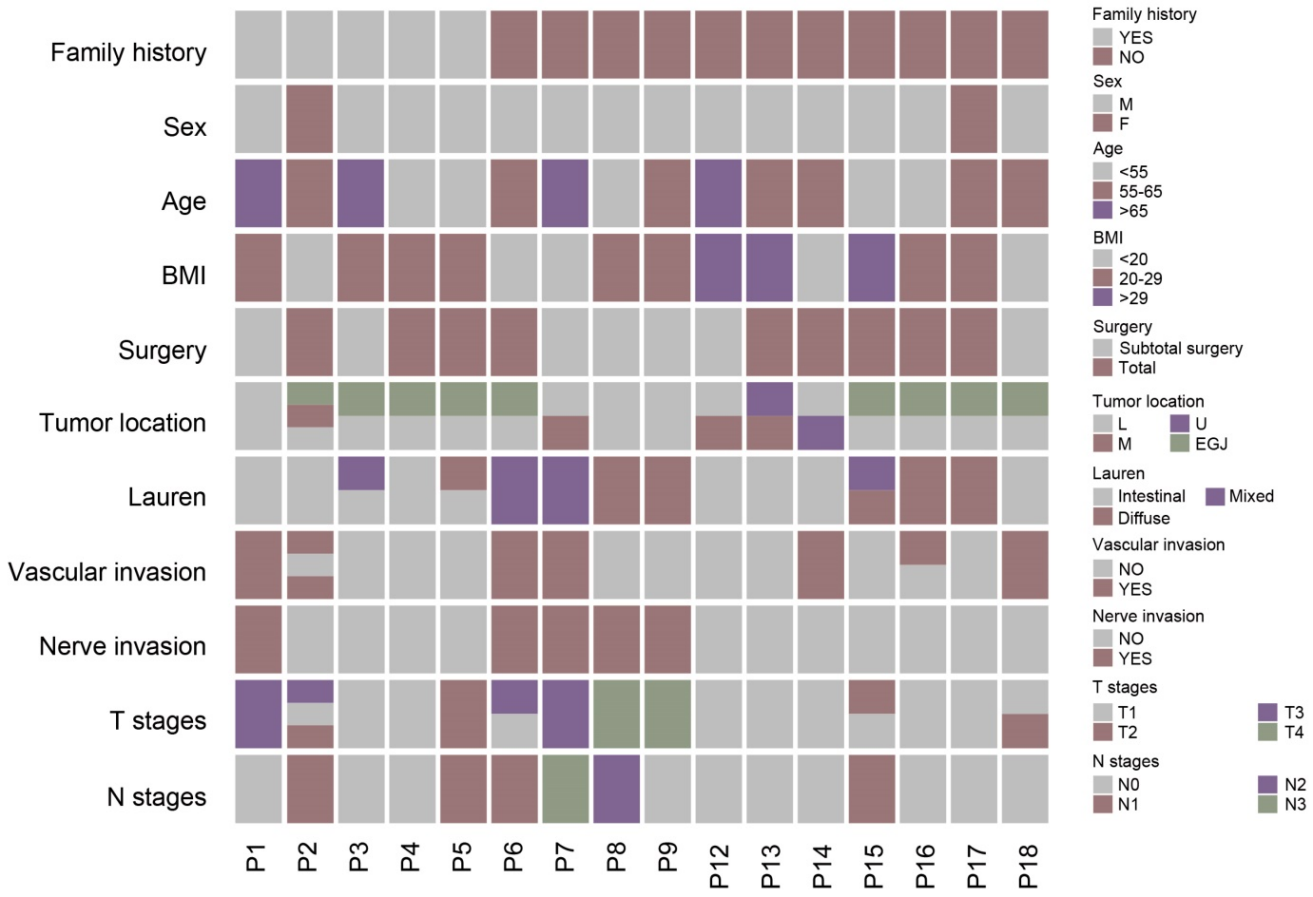
** The clinical-pathological characteristics of multiple gastric cancer.** The different color of columns represents different status of basic information of patients with MGC and pathological characteristics of different tumors of MGC. MGC: multiple gastric cancer.

We included 16 patients with multiple gastric cancer and 33 tumor samples. Samples from two independent tumors were collected from all patients except P2. Male patients were predominant (14 versus 2). The mean age was 59 years. The locations of tumors for each patient were varied, with tumors of 3 patients (P1, P8, and P9) located in a similar region of the stomach, whereas the tumor of the other patients (P2-7 and P12-18) were located in different regions of the stomach. For all samples, most of the tumors (24 samples) were located in the stomach and 9 tumor samples were diagnosed as GC-EGJ. The tumor differentiation of ten patients was identified as identical, however, six other patients including P3, P5 and P15-18, were evaluated as displaying different levels of differentiation. Most of the patients had the same Lauren classification, except P3, P5 and P15. All patients accepted radical surgery, including total or subtotal gastrectomy. Regarding the pathological results, most of patients, except P2 and P16, were classified as having the same lymphatic vessel invasion statement, and all patients were diagnosed with a consistent perineural invasion status. Half of the patients were diagnosed with the same tumor T stage, and the others (P2-4, P6, P13, P15-16, and P18) had different stage tumors. Eighteen tumor samples of all patients were diagnosed as stage T1 and fifteen samples were diagnosed as stage T2-4 tumors (Figure 1). We only observed an *H. pylori* infection in samples of P10. Two patients, P2 and P7, were identified as mismatch repair deficiency (d-MMR).

### Systematic analysis of mutations in MGC tumour samples using WES

We extracted DNA from 33 tumor samples for WES analyses. The average sequencing depth was 266 and 147 for tumor and normal tissue samples, respectively (Supplementary Table 1). Somatic nucleotide variants (SNVs), INDELs, and somatic copy number variants (CNVs) were analyzed for all tumor samples. We identified 519 driver mutations and 34 significantly mutated genes in the MGC samples.

### The mutations identified in MGC have no distinction from that of GC in the TCGA database

**Figure 2:**
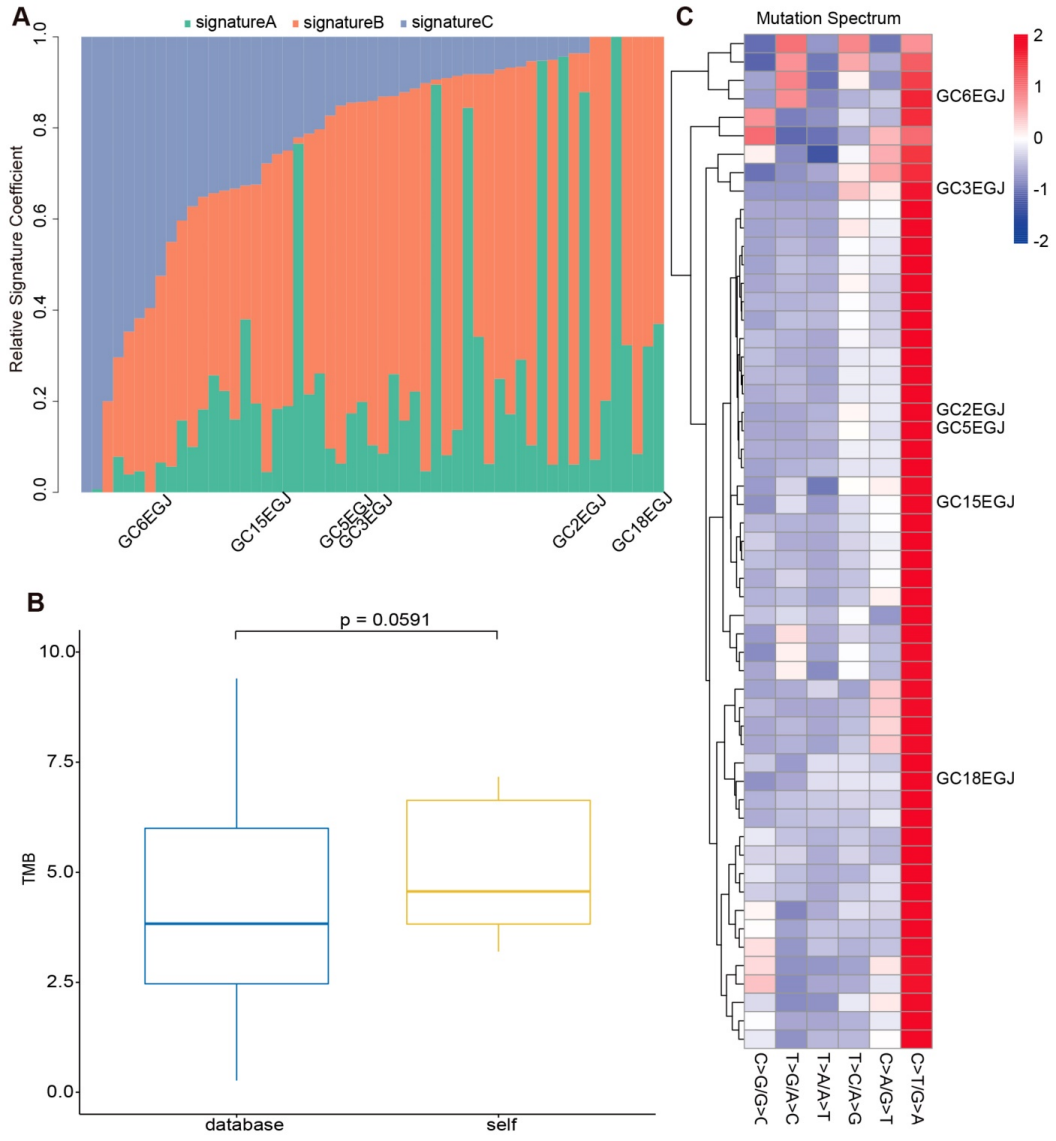
** The comparison on mutations of GC-EGJ between our data and TCGA database.** (A) The column shows the distributions of mutation signatures among GC-EGJ groups from our data and public database. The Y axis represents the fractions of mutation signatures and the X axis indicates the tumor samples, in which the samples with marks are our data and the others are from TCGA database. (B) The box plot depicts the TMB between public data (blue) and our data (yellow). (C) The heatplot represents the mutation spectrum among GC-EGJ groups from our data and public database. The right parts are different samples. Samples with names are from our data and the others from TCGA database. GC-EGJ: esophagogastric junction cancer, TCGA: the cancer genome atlas, TMB: tumor mutation burden

**Figure 3:**
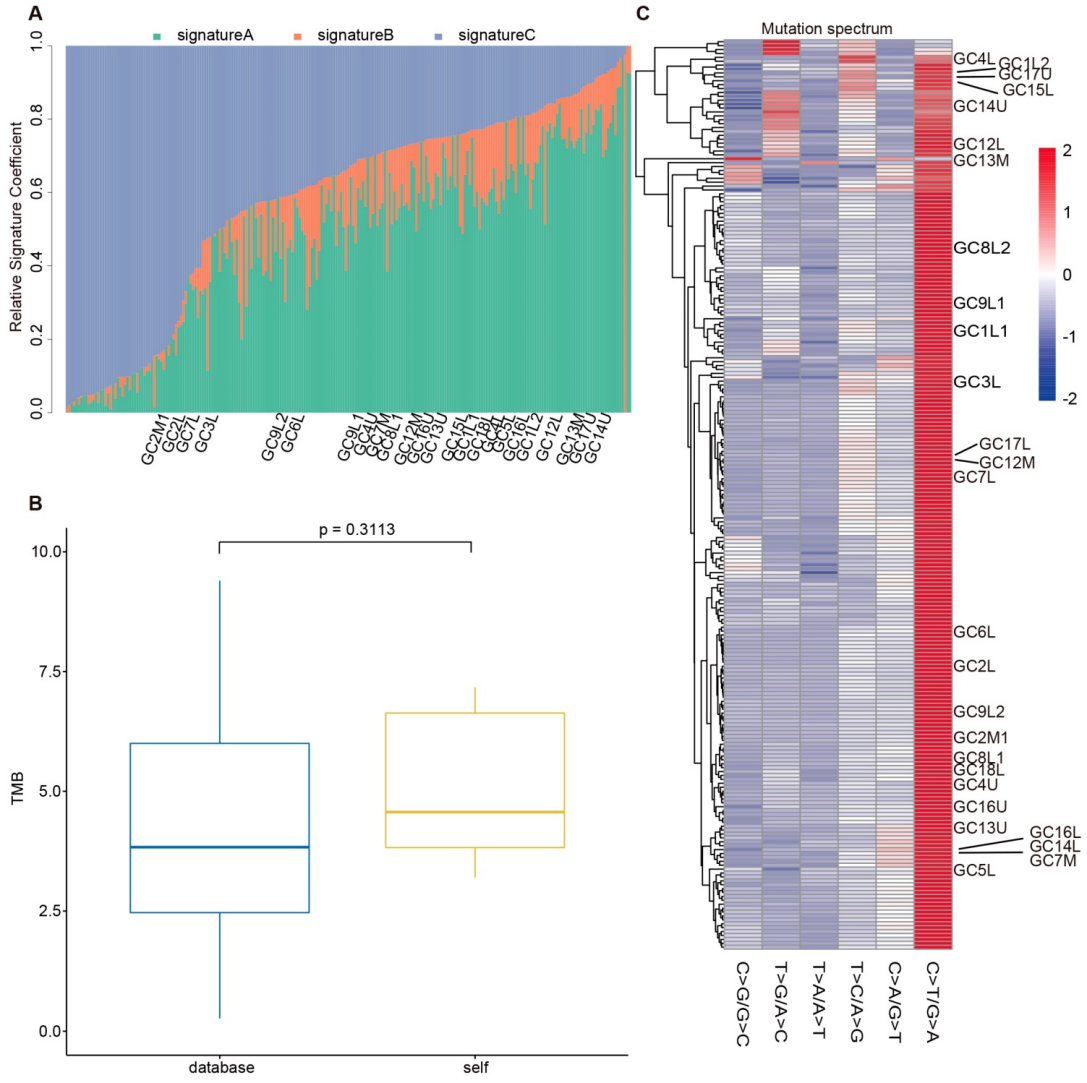
** The comparison on mutations of GC between our data and TCGA database.** (A) The column shows the distributions of mutation signatures among GC groups from our data and public database. (B) The box plot depicts the TMB between public data (blue) and our data (yellow). (C) The heatplot represents the mutation spectrum among GC groups from our data and public database. GC: gastric cancer, TCGA: the cancer genome atlas, TMB: tumor mutation burden

To evaluate the reliability of our sequencing results from the stomach and EGJ, we collected WES data of 208 GCs and 49 GC-EGJs in the TCGA database. We compared the tumor mutation burden (TMB), mutation spectrum and mutation signatures of tumors from different locations between our data and the TCGA database. The difference in the TMB between the samples from the database and our samples in the GC-EGJ groups was not statistically significant (P=0.0591) (Figure 2B). Additionally, the TMB level was also similar in the GC groups between two databases (P=0.3113) (Figure 3B). The mutation spectrum of the GC-EGJ tumors in our study was approximately uniformly distributed within 49 same samples from the public database, with a predominance of the C>T/G>A mutation (Figure 2C). The mutation spectrum of GC in our research was nearly identical to that of 208 samples from the TCGA database and with a similar mutation type to that of GC-EGJ (Figure 3C). The mutation signatures also showed a uniform distribution in the GC and GC-EGJ groups within our data and the TCGA database, respectively (Figure 2A and Figure 3A). However, the predominant mutation signatures were obviously different between the GC and GC-EGJ groups. Signature B predominated in the GC-EGJ groups, but signatures A and C predominated in the GC groups.

The comparative results from our data and the TCGA database indicated the consistent mutations of GC and GC-EGJ with other studies. The GCs and GC-EGJs of patients with MGC have no distinctions from single GC or GC-EGJ.

### The monoclonal and polyclonal relationships of multiple gastric cancer

**Figure 4:**
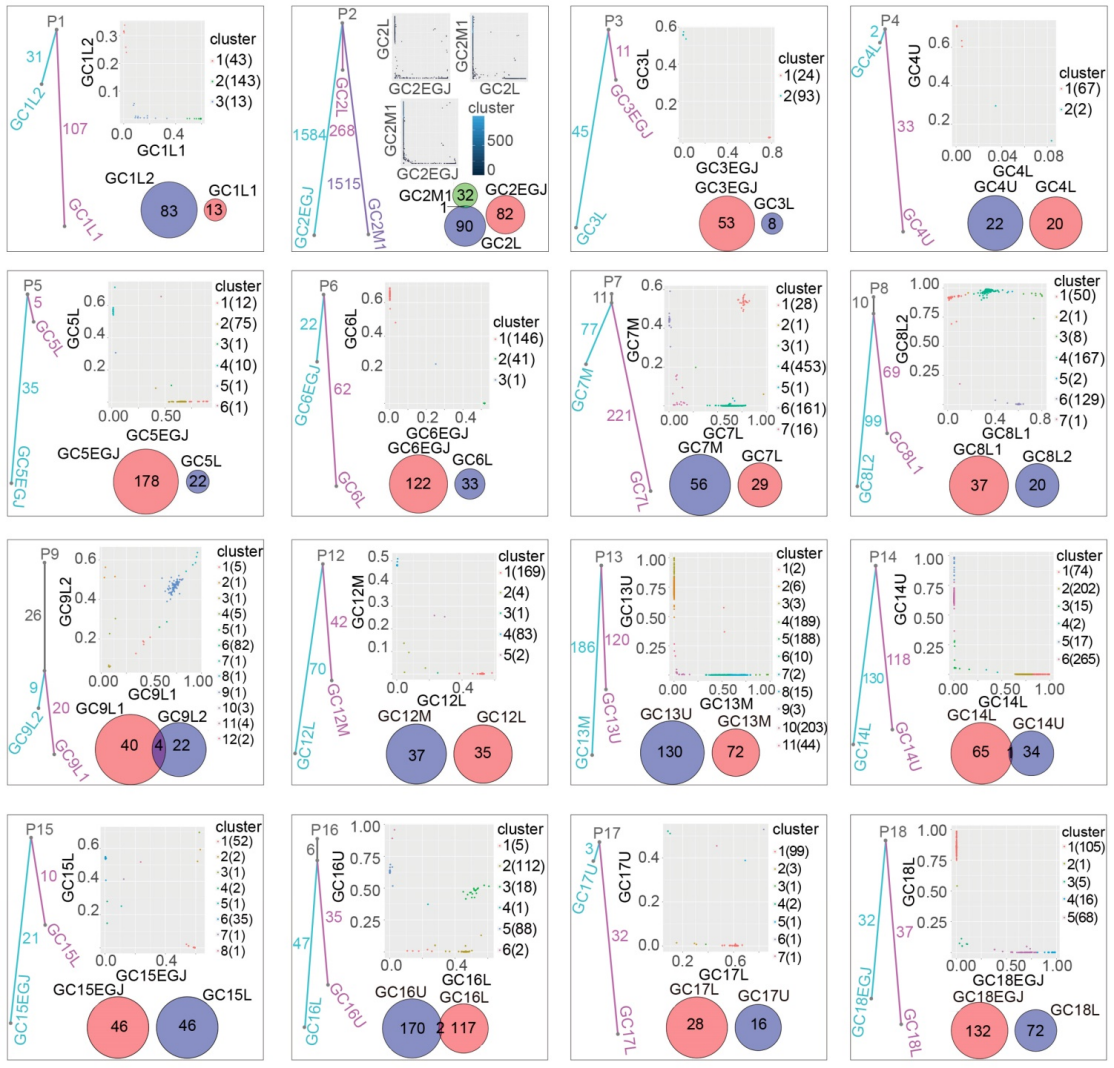
** The clonal relationship of different tumors among MGCs.** The different cube pictures represent different patients from P1 to P18. Fraction of ubiquitous nonsynonymous somatic mutations (trunk) and unique nonsynonymous somatic mutations (branch) in evolutionary trees reveal the relationship of different tumor samples within same patient. In evolutionary trees. Two-dimensional scatter plots show the cancer cell fraction (CCF) of the mutations in different tumor samples. Different clusters were calculated from tumor samples of each MGC patients. Clusters off the axes indicate mutations in all tumor samples. Clusters on the axes reveals mutations in one of tumor samples. Venn diagrams show the relationship of CNVs between different tumor in every MGC patient. The different number represents the number of CNVs numbers for corresponding samples and the overlapped regions are ubiquitous CNVs among different tumor samples from same patients. MGC: multiple gastric cancer, CCF: cancer cell fraction, CNVs: copy number variants

To explore the clonal relationship of tumor samples among patients with multiple gastric cancer, we constructed a phylogeny tree based on the somatic mutations of different samples for each patient (Figure 4). Four of the sixteen had monoclonal tumours, in which 11, 10, 26 and 6 somatic mutations were shared within different tumors in P7, P8, P9 and P16, respectively. However, no common mutation was observed between different tumors from the same patient in the other 12 patients, even though P2 presented thousands of somatic mutations. Therefore, these 12 patients genetically have multiple gastric cancer, and the 4 monoclonal patients may have tumors that metastasized from each other.

To further validate the clonal relationships, we conducted clonal analysis (Figure 4). PyClone analyses take the mutation frequency, copy number variants, tumor purity, loss of heterogeneity and cancer cell fraction into consideration. We identified common subclonal clusters with large numbers of SNVs in a large cancer cell fraction within both tumor components of P7, P8, P9 and P16. However, common clusters with very few SNVs were observed in limited cancer cell fractions from nine patients. No common cluster was observed for P4, P14 and P17. As supplementary evidence, the clonal analysis supported the clonal relationships among multiple gastric cancer samples from P7, P8, P9 and P16. Additionally, the clonal origin was unable to be confirmed strictly using clonal analyses.

In parallel, somatic CNVs were also compared to explore the relationship of different tumors within same patients (Figure 4). We found that only two patients (P9 and P16) shared more than one ubiquitous somatic CNV, in which four and two common CNVs were identified, respectively. However, P2 and P14 only presented one ubiquitous somatic CNV. The other patients were not identified as sharing CNVs among their tumor samples.

In addition, we also compared the SMGs and driver genes within the same patients (Supplementary Figure 1). An *RNF213* splice site mutation was identified between two tumors of P7. *ARID1A* nonsense mutation was detected in both tumors from P8. For P9, we detected common mutations in the significantly mutated genes of *CDH1, KMT2B* and *BAP1*. Although common SMGs were also identified in P2 and P12, the single nucleotide variants of the same SMGs were not identical.

The findings discussed above imply that a part of multiple gastric cancers originates from a common origin, whereas others are polyclonal, indicating genetic difference multiple gastric cancer.

### The role of *MSH2* in the tumorigenesis of multiple gastric cancer

The low probability of carcinogenesis limits the occurrence of multiple tumours in the same organ or patient. The mechanisms underlying the occurrence of MGC and the role of predisposing genes in its occurrence remain obscure.

**Figure 5:**
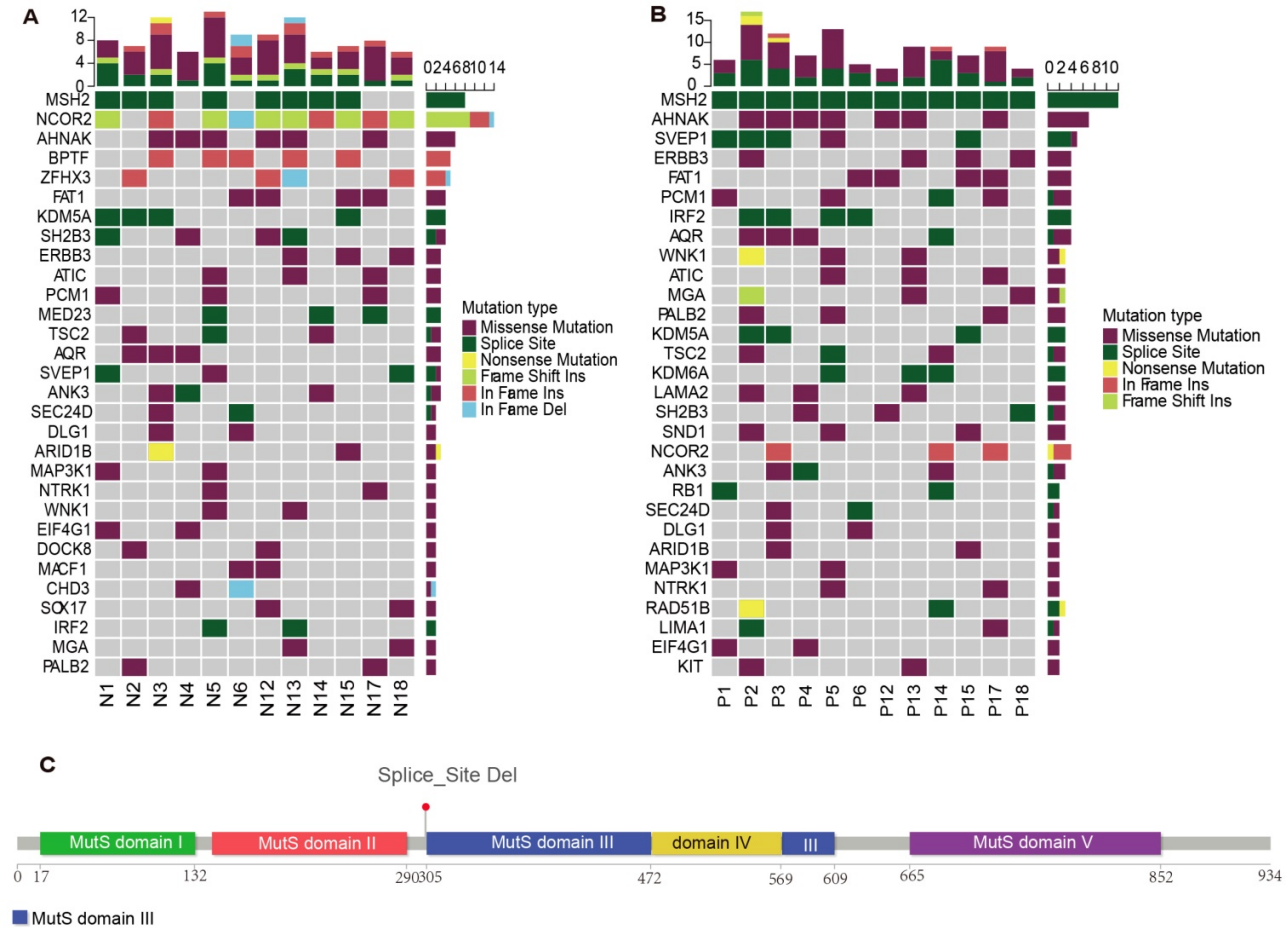
** The germline mutations among genetic MGC patients.** (A) The predisposing genes landscape shows the distribution of germline mutations in MGC patients. The column on top shows the mutational rate of every sample. Heat map shows predisposing genes and mutation type including missense mutation (purple block), splice site (green block), nonsense mutation (yellow block) and so on. (B) The landscape plot shows the distributions of predisposing genes of cancer samples in MGC patients. (C) The figure provides the detailed information about germline mutations of MSH2 gene. The bar chart with different color indicates different protein functional domain. The number below chart represents the length of functional domain. The line means the specific site of mutation. MGC: multiple gastric cancer

To explore the roles of predisposing genes in MGCs, we analyzed the germline mutations of genetic MGCs (Figure 5A). We identified germline predisposing genes by comparing mutations with the Cancer Gene Census (CGC) and two other predisposing gene databases. Germline *MSH2* and *NCOR2* mutations were significantly dominant in 8/12 and 10/12 of patients with genetic MGC, respectively.

Of these mutations, *MSH2 X314*_splice deletion occurred in most of patients, except for P4, P6, P17 and P18. *NCOR2* mutations were identified in most patients, except P2 and P4. Six patients carried nonsense mutations, three patients carried frameshift INDELs and one patient non-frameshift INDELs in this gene. Among the germline *MSH2* mutations identified in MGCs, 40% of patients had a family history of cancer. In addition, germline *AHNAK* missense mutations were detected in 50% of all patients with genetic MGCs.

Among the identified SMGs and driver genes, we found no significant *MSH2, NCOR2* and *AHNAK* mutations in any tumor samples. Therefore, we further analyzed the predisposing genes in the tumor samples (Figure 5B). Interestingly, the *NCOR2* mutations were detected significantly with mutations in only three patients. However, all patients were identified with *MSH2* mutations, including P4, P6, P17 and P18. More interestingly, we found the identical *MSH2* splicing site in all germline and tumor samples (Figure 5C). All germline *AHNAK* mutations were also present in cancer samples from those same patients.

The analyses of germline and cancer predisposing mutations imply the possibility of an important role of predisposing genes, including *MSH2* and *AHNAK*, in the tumorigenesis of MGCs.

### The mutation distributions of genetic multiple gastric cancer

**Figure 6:**
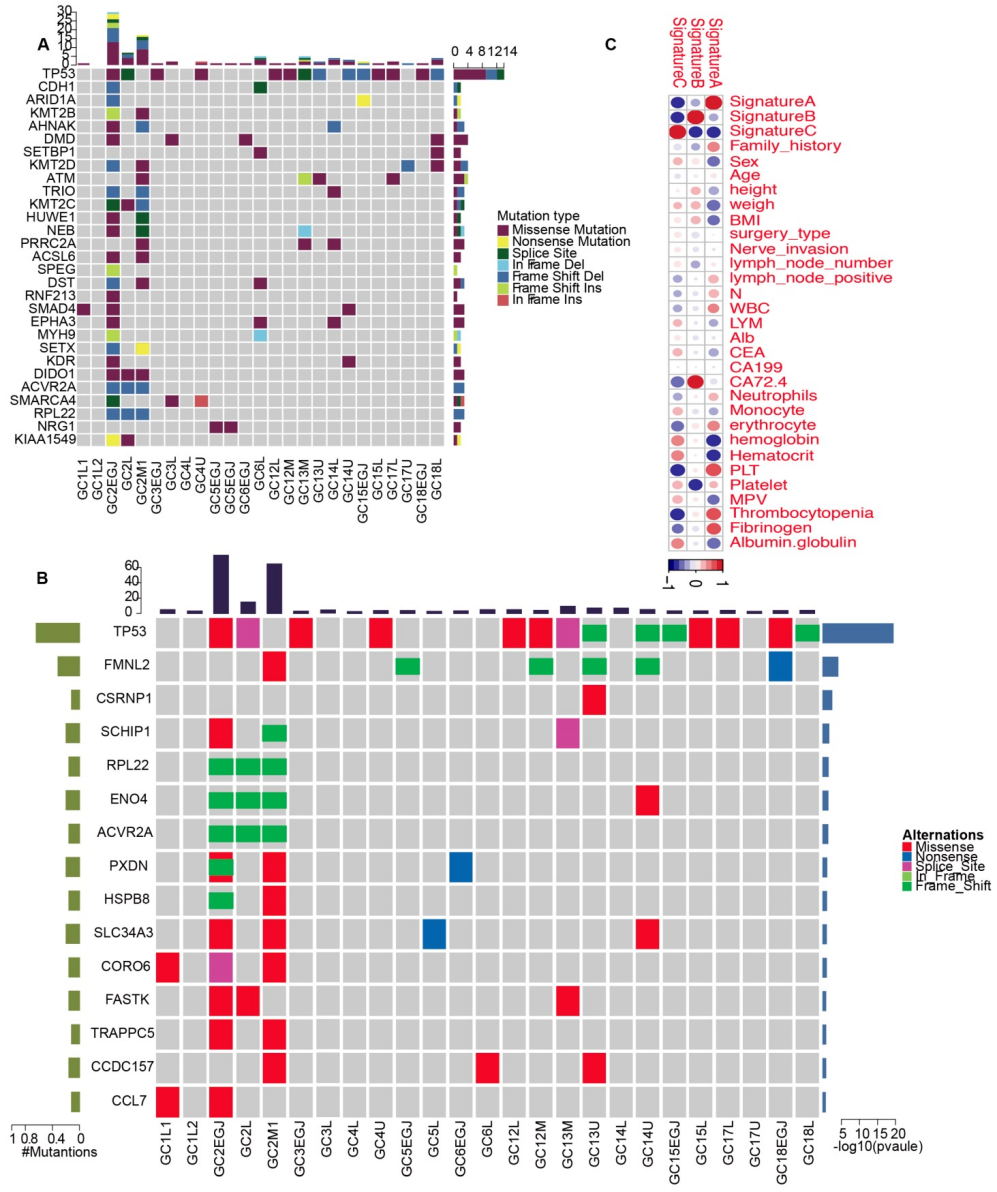
** The driver genes and SMGs landscapes of genetic MGCs.** (A) Driver genes landscape shows the distribution of driver genes among samples of genetic MGCs. The column on top shows the mutation numbers for every sample. Heat map shows the driver genes and mutation type including missense mutation (purple block), splice site (green block), nonsense mutation (yellow block) and so on. (B) SMGs landscape presents the distribution of SMGs among tumor samples of MGCs. (C) The heatplot shows the relationships between clinical characteristics and mutation signatures. The color of each dot represents different extent of association. SMG: significant mutated genes, MGC: multiple gastric cancer

We classified pathologically diagnosed MGCs into monoclonal and polyclonal types, in which the latter represented intrinsic MGC. To comprehensively present the characteristics of mutations in MGCs, we analyzed the SNVs, INDELs, CNVs, mutation spectrum, mutation signatures, SMGs and driver genes for each tumor. On average, 179 SNVs were identified in every MGC tumor sample; however, the average number of SNVs was reduced to 51 after excluding P2. Similarly, an average of 11 frameshift INDELs were detected in each tumor and the value reached 3, except for P2. Patient 2 was very special with 3357 SNVs and six frameshift INDELs. An average of 59 CNVs were observed in each MGC tumor. Regarding the mutation type, the C>T/G>A predominated in all genetic MGCs (Supplementary Figure 2). Most tumors were identified with signature B predominance, however, GC2-EGJ and GC2L were signature A and GC2M with signature C predominance, respectively (Supplementary Figures 3-5). The correlation analysis between clinical characteristics and signatures showed that signature B was associated with the level of CA72.4 (Figure 6C). At the gene level, we identified *TP53* driver mutations including missense mutations and frameshift INDELs in 14 of the 25 tumor samples (Figure 6A and Supplementary Figure 6). In addition, *DMD, KMT2D* and *ATM* driver mutations were also detected in 16% of MGC tumors. From the comprehensive analysis, we only identified *TP53* significantly mutated in 14 of 25 tumor samples. *FMNL2* mutations were found in 6/25 of MGC tumors (Figure 6B and Supplementary Figure 7). We also presented the CNVs for genetic MGCs (Supplementary Figure 8).

In summary, genetic MGCs are characterized by a signature B predominance and a large number of *TP53* mutations.

## Discussion

MGC is a special type of gastric cancer with more than two different tumors at various locations of the stomach. However, the traditional pathology method makes it difficult to diagnose real MGC owing to the lack of the genetic relationship.

Although our study includes few samples, the tumor mutation burden, mutation spectrum, and mutation signatures in the GC and GC-ECJ groups are consistent between our data and the TCGA database. All these findings indicate no specialty between single tumors from MGC and solitary GC.

In our study, we conducted WES to explore the genetic links at different levels. We found that some MGCs shared more than 2 somatic mutations among tumors within each patient. However, other MGCs have no common somatic mutations among samples. The CNVs analysis and clonal analysis also supported similar results. The PyClone analysis identified a ubiquitous subclonal cluster in P7-9 and P16 exhibited a common cluster in a large cancer cell fraction within both tumor components, which might support the monoclonal origin of these MGCs 19-21. In the CNV analysis, P9 and P16 were also found to share more than 2 somatic CNVs and only one ubiquitous CNV was identified in P2 and P14. These results could only support the monoclonality of P9 and P16 rather than P2 and P14. The chance for the occurrence of one common CNV within two different samples is theoretically possible. However, the probability becomes extremely low when more than two CNVs are identified between two samples. Although the CNV analysis using WES and WGS is similar 22, we also regard it as supplementary evidence due to its limitations. Based on the findings from our study, MGCs should be classified as genetic and metastatic MGCs, indicating different mechanisms of carcinogenesis. Although we still did not detect cancer tissues within the normal mucosa between tumors of metastatic MGCs, we are unable to deny the genetic links indicating their common origin. We carefully re-evaluated the pathological diagnosis of each tumor sample from P7-9 and P16, the T3 and T4 stage tumor from P7-9 may contribute to their metastasis within tumors. The positive lymphatic vessel invasion of P7 and P16 may have led to the development of multiple tumours in these patients.

Another puzzle is why tumors occur in the same patient. We found that some germline *MSH2* mutations may contribute to the susceptibility of carcinogenesis. Owing to the lack of *MSH2* mutations in MGCs, we conducted germline and cancer predisposing genes analyses. Surprisingly, *MSH2* mutations were identified in most of patients with MGCs. As we all know, *MSH2* is a widely known gene participating in the repair during DNA replication. It is also associated with the occurrence of Lynch syndrome 23-25. The syndrome is often accompanied by a high incidence of colorectal cancer, endometrial cancer, gastric cancer and other tumors 26. Of which, the incidence of gastric cancer reaches 9% for patients with germline *MSH2* mutations 23. Interestingly, 33.3% of patients with genetic MGC have an obvious family history of cancer. Another intriguing case is that P2 was diagnosed with colon cancer a few years ago before occurrence of gastric cancer. Therefore, a reasonable speculation is that the germline *MSH2* mutations may contribute to the occurrence of multiple tumors in the stomach.

The treatment modalities for MGCs are similar to typical GCs. For MGCs at different stages, clinicians may select surgery, chemotherapy and radiotherapy. However, researchers have not clearly determined whether the special mechanisms of tumorigenesis are associated with key factors contributing to the treatment of MGCs. The first question is the appropriate surgery for MGCs. As we all know, Moertel 5 proposes that surgeons should properly expand the extent of surgery for MGCs. The guideline also recommends prophylactic gastrectomy for young asymptomatic persons in families with a history of highly penetrant hereditary diffuse gastric cancer 27, 28. Additionally, prophylactic surgery has also been strongly recommended for many types of tumors such as breast cancer and ovarian cancer with *BRAC1* or *BRAC2* germline mutations 29, 30. As shown in the present study, 66.7% of genetic MGCs were associated with a germline *MSH2* mutation, which may make them susceptible to genetic MGC. Further studies are needed to determine whether a more positive surgery strategy should be employed for genetic MGC. Our evidence is not sufficient to change the surgical strategy for patients with genetic MGC, and additional prospective trials are warranted. Germline *MSH2* mutations may play an important role not only in the carcinogenesis of genetic MGCs but also in solitary gastric cancer with family history. Therefore, subtotal and local resection may be reconsidered for these specific patients. Endoscopic submucosa dissection is recommended for patients with gastric cancer at an early stage. However, the patients with a high risk of lymph node metastasis should undergo radical surgery. Therefore, patients with metastatic MGC patients should receive radical surgery even when they are diagnosed at early stages. Another question is the value of immunotherapy for genetic MGC. As the most promising treatment modality for cancer, immunotherapy, especially immune checkpoint inhibitors, have an important effect on the cancer treatment. Although the PD-L1 for all genetic MGCs is negative, the germline and cancer *MSH2* mutations may also make anti-PD-1/PD-L1 therapy a very significant modality for MGC 31-33.

## Conclusions

Whole-exome sequencing analyses suggest the monoclonal and polyclonal origins of MGC, which may promote the classification of MGC into genetic and metastatic MGC. For patients with genetic MGC, germline *MSH2 X314*_splice variants may contribute to carcinogenesis, prompting the consideration of a more radical surgery and/or anti-PD-1/PD-L1 therapy therapy.

## Supplementary Material

Supplementary materials and methods, figures and table.

## Abbreviations

MGC: multiple gastric cancer
GC: gastric cancer
GC-EGJ: esophagogastric junction cancer
WES: whole-exome sequencing
SNV: somatic nucleotide variant
CNV: copy number variation
SMG: significantly mutated gene

## References

[1] Bray F, Ferlay J, Soerjomataram I, Siegel RL, Torre LA, Jemal A (2018). Global cancer statistics 2018: GLOBOCAN estimates of incidence and mortality worldwide for 36 cancers in 185 countries. CA: a cancer journal for clinicians.

[2] Chen W, Zheng R, Zhang S, Zeng H, Zuo T, Xia C (2017). Cancer incidence and mortality in China in 2013: an analysis based on urbanization level. Chinese journal of cancer research = Chung-kuo yen cheng yen chiu.

[3] Morgagni P, Marfisi C, Gardini A, Marrelli D, Saragoni L, Roviello F (2009). Subtotal gastrectomy as treatment for distal multifocal early gastric cancer. Journal of gastrointestinal surgery: official journal of the Society for Surgery of the Alimentary Tract.

[4] Yoo JH, Shin SJ, Lee KM, Choi JM, Wi JO, Kim DH (2013). How can we predict the presence of missed synchronous lesions after endoscopic submucosal dissection for early gastric cancers or gastric adenomas?. Journal of clinical gastroenterology.

[5] Moertel CG, Bargen JA, Soule EH (1957). Multiple gastric cancers; review of the literature and study of 42 cases. Gastroenterology.

[6] Kim JH, Jeong SH, Yeo J, Lee WK, Chung DH, Kim KO (2016). Clinicopathologic Similarities of the Main and Minor Lesions of Synchronous Multiple Early Gastric Cancer. Journal of Korean medical science.

[7] Eom BW, Lee JH, Choi IJ, Kook MC, Nam BH, Ryu KW (2012). Pretreatment risk factors for multiple gastric cancer and missed lesions. Journal of surgical oncology.

[8] Kim HM, Kim HK, Lee SK, Cho JH, Pak KH, Hyung WJ (2012). Multifocality in early gastric cancer does not increase the risk of lymph node metastasis in a single-center study. Annals of surgical oncology.

[9] Borie F, Plaisant N, Millat B, Hay JM, Fagniez PL, De Saxce B (2003). Treatment and prognosis of early multiple gastric cancer. European journal of surgical oncology: the journal of the European Society of Surgical Oncology and the British Association of Surgical Oncology.

[10] Otsuji E, Kuriu Y, Ichikawa D, Okamoto K, Hagiwara A, Yamagishi H (2005). Clinicopathologic characteristics and prognosis of synchronous multifocal gastric carcinomas. American journal of surgery.

[11] Hansford S, Kaurah P, Li-Chang H, Woo M, Senz J, Pinheiro H (2015). Hereditary Diffuse Gastric Cancer Syndrome: CDH1 Mutations and Beyond. JAMA oncology.

[12] Sahasrabudhe R, Lott P, Bohorquez M, Toal T, Estrada AP, Suarez JJ (2017). Germline Mutations in PALB2, BRCA1, and RAD51C, Which Regulate DNA Recombination Repair, in Patients With Gastric Cancer. Gastroenterology.

[13] Slavin T, Neuhausen SL, Rybak C, Solomon I, Nehoray B, Blazer K (2017). Genetic Gastric Cancer Susceptibility in the International Clinical Cancer Genomics Community Research Network. Cancer genetics.

[14] Tedaldi G, Pirini F, Tebaldi M, Zampiga V, Cangini I, Danesi R (2019). Multigene Panel Testing Increases the Number of Loci Associated with Gastric Cancer Predisposition. Cancers.

[15] Huntsman DG, Carneiro F, Lewis FR, MacLeod PM, Hayashi A, Monaghan KG (2001). Early gastric cancer in young, asymptomatic carriers of germ-line E-cadherin mutations. The New England journal of medicine.

[16] Carter SL, Cibulskis K, Helman E, McKenna A, Shen H, Zack T (2012). Absolute quantification of somatic DNA alterations in human cancer. Nature biotechnology.

[17] Roth A, Khattra J, Yap D, Wan A, Laks E, Biele J (2014). PyClone: statistical inference of clonal population structure in cancer. Nature methods.

[18] Integrated genomic characterization of oesophageal carcinoma Nature. 2017; 541: 169-75.

[19] Wang A, Wu L, Lin J, Han L, Bian J, Wu Y (2018). Whole-exome sequencing reveals the origin and evolution of hepato-cholangiocarcinoma. Nature communications.

[20] Zhang J, Fujimoto J, Zhang J, Wedge DC, Song X, Zhang J (2014). Intratumor heterogeneity in localized lung adenocarcinomas delineated by multiregion sequencing. Science.

[21] Miao R, Luo H, Zhou H, Li G, Bu D, Yang X (2014). Identification of prognostic biomarkers in hepatitis B virus-related hepatocellular carcinoma and stratification by integrative multi-omics analysis. J Hepatol.

[22] Xue R, Li R, Guo H, Guo L, Su Z, Ni X (2016). Variable Intra-Tumor Genomic Heterogeneity of Multiple Lesions in Patients With Hepatocellular Carcinoma. Gastroenterology.

[23] Rahner N, Steinke V, Schlegelberger B, Eisinger F, Hutter P, Olschwang S (2013). Clinical utility gene card for: Lynch syndrome (MLH1, MSH2, MSH6, PMS2, EPCAM) - update 2012. European journal of human genetics: EJHG.

[24] Latham A, Srinivasan P, Kemel Y, Shia J, Bandlamudi C, Mandelker D (2019). Microsatellite Instability Is Associated With the Presence of Lynch Syndrome Pan-Cancer. Journal of clinical oncology: official journal of the American Society of Clinical Oncology.

[25] Tricarico R, Kasela M, Mareni C, Thompson BA, Drouet A, Staderini L (2017). Assessment of the InSiGHT Interpretation Criteria for the Clinical Classification of 24 MLH1 and MSH2 Gene Variants. Human mutation.

[26] Sinicrope FA (2018). Lynch Syndrome-Associated Colorectal Cancer. The New England journal of medicine.

[27] Hebbard PC, Macmillan A, Huntsman D, Kaurah P, Carneiro F, Wen X (2009). Prophylactic total gastrectomy (PTG) for hereditary diffuse gastric cancer (HDGC): the Newfoundland experience with 23 patients. Annals of surgical oncology.

[28] Seevaratnam R, Coburn N, Cardoso R, Dixon M, Bocicariu A, Helyer L (2012). A systematic review of the indications for genetic testing and prophylactic gastrectomy among patients with hereditary diffuse gastric cancer. Gastric cancer: official journal of the International Gastric Cancer Association and the Japanese Gastric Cancer Association.

[29] Hartmann LC, Schaid DJ, Woods JE, Crotty TP, Myers JL, Arnold PG (1999). Efficacy of bilateral prophylactic mastectomy in women with a family history of breast cancer. The New England journal of medicine.

[30] Grann VR, Jacobson JS, Whang W, Hershman D, Heitjan DF, Antman KH (2000). Prevention with tamoxifen or other hormones versus prophylactic surgery in BRCA1/2-positive women: a decision analysis. The cancer journal from Scientific American.

[31] Le DT, Durham JN, Smith KN, Wang H, Bartlett BR, Aulakh LK (2017). Mismatch repair deficiency predicts response of solid tumors to PD-1 blockade. Science (New York, NY).

[32] Long J, Lin J, Wang A, Wu L, Zheng Y, Yang X (2017). PD-1/PD-L blockade in gastrointestinal cancers: lessons learned and the road toward precision immunotherapy. Journal of hematology & oncology.

[33] Broos K, Lecocq Q, Raes G, Devoogdt N, Keyaerts M, Breckpot K (2018). Noninvasive imaging of the PD-1:PD-L1 immune checkpoint: Embracing nuclear medicine for the benefit of personalized immunotherapy. Theranostics.

