# Supplementary material for: Molecular characteristics of synchronous multiple gastric cancer
Source: Theranostics. 2020 Apr 7;10(12):5489–500. doi: 10.7150/thno.42814 (PMC7196298; doi:10.7150/thno.42814)
Supplement: Supplementary file 1 — Supplementary materials and methods, figures and table. [file thnov10p5489s1.pdf]

Materials and methods

Patients and tissue samples

We collected 16 multiple gastric cancer patients who underwent subtotal and total gastrectomy at Peking Cancer Hospital from January 2016 to December 2017. Thirty- three tumor samples and sixteen normal gastric tissue or blood samples were obtained for experiment. The clinical information for every patient was collected in detail and the pathological diagnosis for every tumor tissues was confirmed again by two independent pathologists. We perform HE staining for selections of every tumor from multiple gastric cancer patients. Informed consent was obtained for every patient. The study protocol was approved by the ethical committee of Peking Cancer Hospital.

DNA collection and whole-exome sequencing

DNA Quantification & Qualification

The quality of isolated genomic DNA was verified by using these two methods in combination:

(1) DNA degradation and contamination were monitored on 1% agarose gels.

(2) DNA concentration was measured by Qubit® DNA Assay Kit in Qubit® 2.0 Fluorometer (Invitrogen, USA).

Library preparation

A total amount of 0.6 μg genomic DNA per sample was used as input material for the DNA sample preparation. Sequencing libraries were generated using Agilent SureSelect Human All Exon V6 kit (Agilent Technologies, CA, USA) following manufacturer's recommendations and index codes were added to each sample

Briefly, fragmentation was carried out by hydrodynamic shearing system (Covaris, Massachusetts, USA) to generate 180-280 bp fragments. Remaining sticky ends were converted into blunt ends via exonuclease/polymerase activities. After adenylation of 3' ends of DNA fragments, adapter oligonucleotides were ligated. DNA fragments with ligated adapter molecules on both ends were selectively enriched in a PCR reaction. After PCR reaction, libraries were hybridized in liquid phase with biotin labeled probe, then magnetic beads with streptomycin were used to capture the exons of genes. Captured libraries were enriched in a PCR reaction to add index tags to prepare for sequencing. Products were purified using AMPure XP system (Beckman Coulter, Beverly, USA) and quantified using the Agilent high sensitivity DNA assay on the Agilent Bioanalyzer 2100 system.

Clustering & Sequencing

The clustering of the index-coded samples was performed on a cBot Cluster Generation System using Hiseq PE Cluster Kit (Illumina) according to the manufacturer's instructions. After cluster generation, the DNA libraries were sequenced on Illumina Hiseq platform and 150 bp paired-end reads were generated.

Bioinformatics Analysis Pipeline

1.Basic bioinformatics analysis

Quality Control

The original fluorescence image files obtained from Hiseq platform are transformed to short reads (Raw data) by base calling and these short reads are recorded in FASTQ format, which contains sequence information and corresponding sequencing quality information.

Sequence artifacts, including reads containing adapter contamination, low-quality nucleotides and unrecognizable nucleotide (N), undoubtedly set the barrier for the subsequent reliable bioinformatics analysis. Hence quality control is an essential step and was applied to guarantee the significant downstream analysis.

The steps of data processing were as follows:

(1) Discard a paired read if either one read contains adapter contamination (>10 nucleotides aligned to the adapter, allowing ≤ 10% mismatches);

(2) Discard a paired read if more than 10% of bases are uncertain in either one read;

(3) Discard a paired read if the proportion of low quality (Phred quality <5) bases is over 50% in either one read.

All the downstream bioinformatics analyses were based on the high quality clean data, which were retained after these steps. At the same time, QC statistics including total reads number, raw data, raw depth, sequencing error rate and percentage of reads with Q30 (the percent of bases with phred-scaled quality scores greater than 30) were calculated and summarized.

Reads Mapping to Reference Sequence

Valid sequencing data were mapped to the reference human genome (UCSC hg19) by Burrows-Wheeler Aligner (BWA) software[1] to get the original mapping results stored in BAM format. If one or one paired read(s) were mapped to multiple positions, the strategy adopted by BWA was to choose the most likely placement. If two or more most likely placements presented, BWA picked one randomly. Then, SAMtools[2] and Picard (http://broadinstitute.github.io/picard/) were used to sort BAM files and do duplicate marking, local realignment, and base quality recalibration to generate final BAM file for computation of the sequence coverage and depth. Mapping step was very difficult due to mismatches, including true mutation and sequencing error, and duplicates resulted from PCR amplification. These duplicate reads were uninformative and should not be considered as evidence for variants. These duplicate reads were uninformative and were not considered as evidence for variants. We used Picard to mark these duplicates for follow up analysis.

Variant Calling

SAMtools[2] mpileup and bcftools were used to do variant calling and identify SNP, InDels.

Functional Annotation

Functional annotation was very important because the link between genetic variations and diseases were clarified in this step. ANNOVAR[3] was performed to do annotation for VCF (Variant Call Format) obtained in the previous effort. dbSNP[4], 1000 Genome[5] and other related existing databases were applied to characterize the detected variants. Exonic variants, gene transcript annotation databases, such as Consensus CDS, RefSeq, Ensembl and UCSC[6], were also included to determine amino acid alternation. Annotation content contained the variant position, variant type, conservative prediction, *etc.* These annotation results helped to locate-causing mutations.

Somatic Mutation Calling

The somatic SNVs were detected by muTect [7] and the somatic InDel by Strelka [8]. Control-FREEC was used to detect somatic CNVs[9].

Mutation spectrum and mutation signature

96 trinucleotides of exonic point mutations were extracted, and nonnegative matrix factorization (NMF) algorithm[10] was used to decipher the underline mutation signatures[11]. Signatures A, B, and C were identified in tumor samples and the signature spectrums were also analyzed. These inferred signatures were compared to the catalog of 30 consensus signatures that were described on the COSMIC website[11] (https://cancer.sanger.ac.uk/cosmic/signatures).

2. Advanced bioinformatics analysis

In order to explore the relationship of different tumor samples within each patient, we conducted clonal analysis, phylogenetic trees construction and comparisons between CNVs, driver genes and significantly mutated genes.

Phylogenetic trees construction

Phylogenetic trees were constructed at the somatic state. Branch lengths were proportional to the number of mutations separating the branching points.

Clonal frequency analysis

Pyclone (version 0.12.7) was used to evaluate the clonal population structures. Allelic frequencies of selected somatic mutations were obtained using the number of reads covered and the number of reads carrying a variation. The copy number value at each of these loci was obtained from Control-FREEC[9], and the tumor content was inferred by Absolute[12]. PyClone[13] used a hierarchical Bayesian model to calculate the clonal frequency for each mutation, and MCMC analysis was applied for clonal population clustering and cellular frequency inference.

Significantly Mutated Genes (SMGs)

Compared to the background mutation rate, MuSiC[14] （Music-0.04） package applies convolution test (CT) to summarize p-values of mutational significance. Genes were considered to be SMGs if at least Q-value < 0.2. For SMGs, we conducted pathway enrichment analyses with PathScan software[15]. Databases such as KEGG[16], Biocarta, PID and Teactome were utilized to perform this analysis

Identification of Driver gene

In order to find the tumor drive genes, we compared the four databases including CGC[17], Bert Vogelstein[18], SMG127[19] and comprehensive database[20] with an in-house script.

3. The comparisons between self and public data

To evaluate our sequencing results from stomach and EGJ, we collected WES data of 208 GCs and 49 GCs-EGJ from TCGA database, respectively. We conducted comparison of tumor mutation burden (TMB)， mutation spectrum and mutation signatures of different location tumors among our data and TCGA database.

Tumor Mutational Burden （TMB）

Tumor Mutational Burden was calculated for included tumor samples and the TCGA samples[21]. The TMB was calculated separately by tumor site including the esophagus and stomach samples. The boxplot shows the distribution of the TMB with boxplot package.

Mutation spectrum comparisons

The distributions of six mutation types within our sequencing data and TCGA samples were presented and compared.

Mutation signatures comparisons

Similarly, the spectrum of identified signature A, B and C was compared between our data and public data. The heatmap was used to depict the distribution of them.

The identification of predisposing genes

To explore the roles of predisposing genes playing in the MGCs, we analyzed the germline mutations of cancer predisposing genes in genetic MGCs. The germline mutations identified in cancer-predisposing genes were compared with CGC database[17].

The study of molecular characteristics of genetic MGCs

To further study the molecular characteristics of genetic MGCs, we re-analyzed the mutation spectrum, mutation signatures, driver mutations, SMGs, clonal analysis and CNV analysis of genetic MGC tumor samples. We also use corrplot R package to calculate mutation characteristics of all the samples and the correlation between clinical information.

Reference

1 Li, H. & Durbin, R. Fast and accurate short read alignment with Burrows-Wheeler transform. *Bioinformatics (Oxford, England)* **25**, 1754-1760, doi:10.1093/bioinformatics/btp324 (2009).

2 Li, H. *et al.* The Sequence Alignment/Map format and SAMtools. *Bioinformatics (Oxford, England)* **25**, 2078-2079, doi:10.1093/bioinformatics/btp352 (2009).

3 Wang, K., Li, M. & Hakonarson, H. ANNOVAR: functional annotation of genetic variants from high-throughput sequencing data. *Nucleic acids research* **38**, e164, doi:10.1093/nar/gkq603 (2010).

4 Sherry, S. T. *et al.* dbSNP: the NCBI database of genetic variation. *Nucleic acids research* **29**, 308-311 (2001).

5 Abecasis, G. R. *et al.* An integrated map of genetic variation from 1,092 human genomes. *Nature* **491**, 56-65, doi:10.1038/nature11632 (2012).

6 Kent, W. J. *et al.* The human genome browser at UCSC. *Genome research* **12**, 996-1006, doi:10.1101/gr.229102 (2002).

7 Cibulskis, K. *et al.* Sensitive detection of somatic point mutations in impure and heterogeneous cancer samples. *Nature biotechnology* **31**, 213-219, doi:10.1038/nbt.2514 (2013).

8 Saunders, C. T. *et al.* Strelka: accurate somatic small-variant calling from sequenced tumor-normal sample pairs. *Bioinformatics* **28**, 1811-1817, doi:10.1093/bioinformatics/bts271 (2012).

9 Boeva, V. *et al.* Control-FREEC: a tool for assessing copy number and allelic content using next-generation sequencing data. *Bioinformatics (Oxford, England)* **28**, 423-425, doi:10.1093/bioinformatics/btr670 (2012).

10 Alexandrov, L. B., Nik-Zainal, S., Wedge, D. C., Campbell, P. J. & Stratton, M. R. Deciphering signatures of mutational processes operative in human cancer. *Cell reports* **3**, 246-259, doi:10.1016/j.celrep.2012.12.008 (2013).

11 Forbes, S. A. *et al.* COSMIC: mining complete cancer genomes in the Catalogue of Somatic Mutations in Cancer. *Nucleic acids research* **39**, D945-950, doi:10.1093/nar/gkq929 (2011).

12 Carter, S. L. *et al.* Absolute quantification of somatic DNA alterations in human cancer. *Nature biotechnology* **30**, 413-421, doi:10.1038/nbt.2203 (2012).

13 Roth, A. *et al.* PyClone: statistical inference of clonal population structure in cancer. *Nature methods* **11**, 396-398, doi:10.1038/nmeth.2883 (2014).

14 Dees, N. D. *et al.* MuSiC: identifying mutational significance in cancer genomes. *Genome research* **22**, 1589-1598, doi:10.1101/gr.134635.111 (2012).

15 Wendl, M. C. *et al.* PathScan: a tool for discerning mutational significance in groups of putative cancer genes. *Bioinformatics (Oxford, England)* **27**, 1595-1602, doi:10.1093/bioinformatics/btr193 (2011).

16 Kanehisa, M. & Goto, S. KEGG: kyoto encyclopedia of genes and genomes. *Nucleic acids research* **28**, 27-30 (2000).

17 Futreal, P. A. *et al.* A census of human cancer genes. *Nature reviews. Cancer* **4**, 177-183, doi:10.1038/nrc1299 (2004).

18 Vogelstein, B. *et al.* Cancer genome landscapes. *Science (New York, N.Y.)* **339**, 1546-1558, doi:10.1126/science.1235122 (2013).

19 Kandoth, C. *et al.* Mutational landscape and significance across 12 major cancer types. *Nature* **502**, 333-339, doi:10.1038/nature12634 (2013).

20 Tamborero, D. *et al.* Comprehensive identification of mutational cancer driver genes across 12 tumor types. *Scientific reports* **3**, 2650, doi:10.1038/srep02650 (2013).

21 Integrated genomic characterization of oesophageal carcinoma. *Nature* **541**, 169-175, doi:10.1038/nature20805 (2017).

Supplementary Figure 1: The comparison of driver genes and SMGs within different tumor samples of same patients

Legend: (A) Driver genes landscape shows the distribution of driver genes of all tumor samples. The x-axis shows the different samples of same patients. (B) SMGs landscape shows the distribution of SMGs of all included samples.

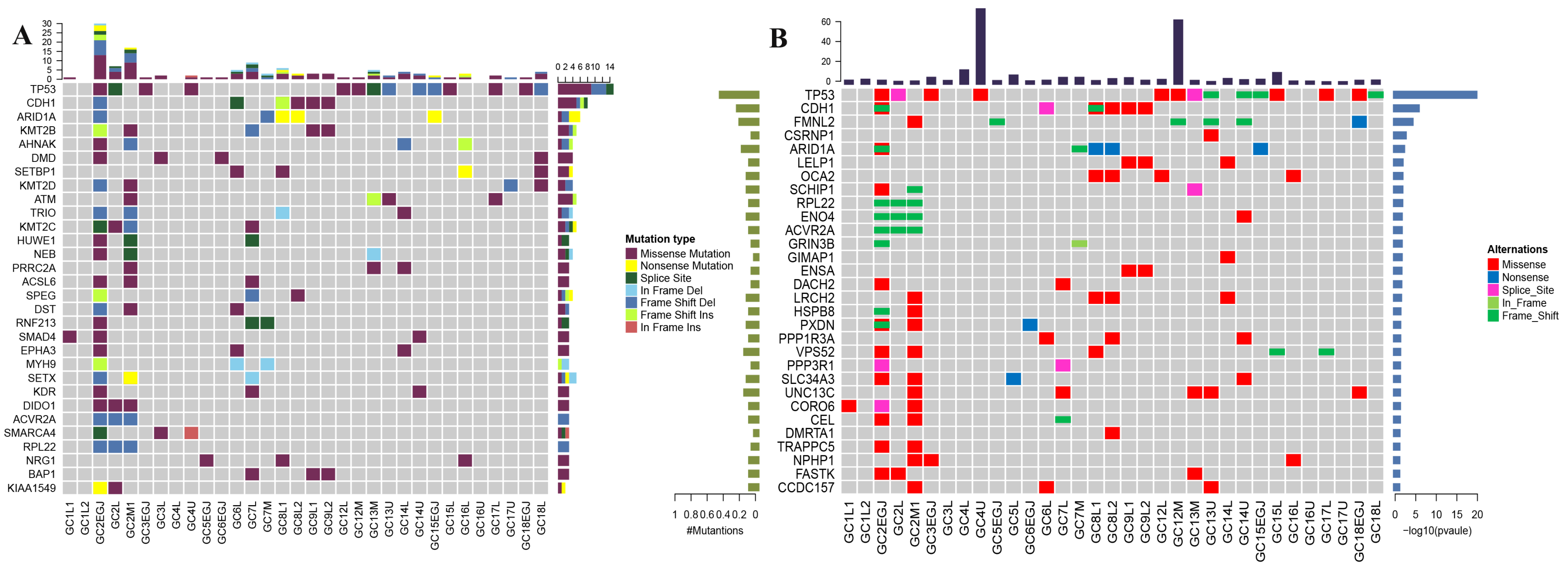

Supplementary Figure 2: The mutation spectrum of genetic MGCs.

Legend: The heatplot shows the mutation spectrum within all genetic MGC tumor samples. The color represents the proportion of each mutation type in different tumor samples.

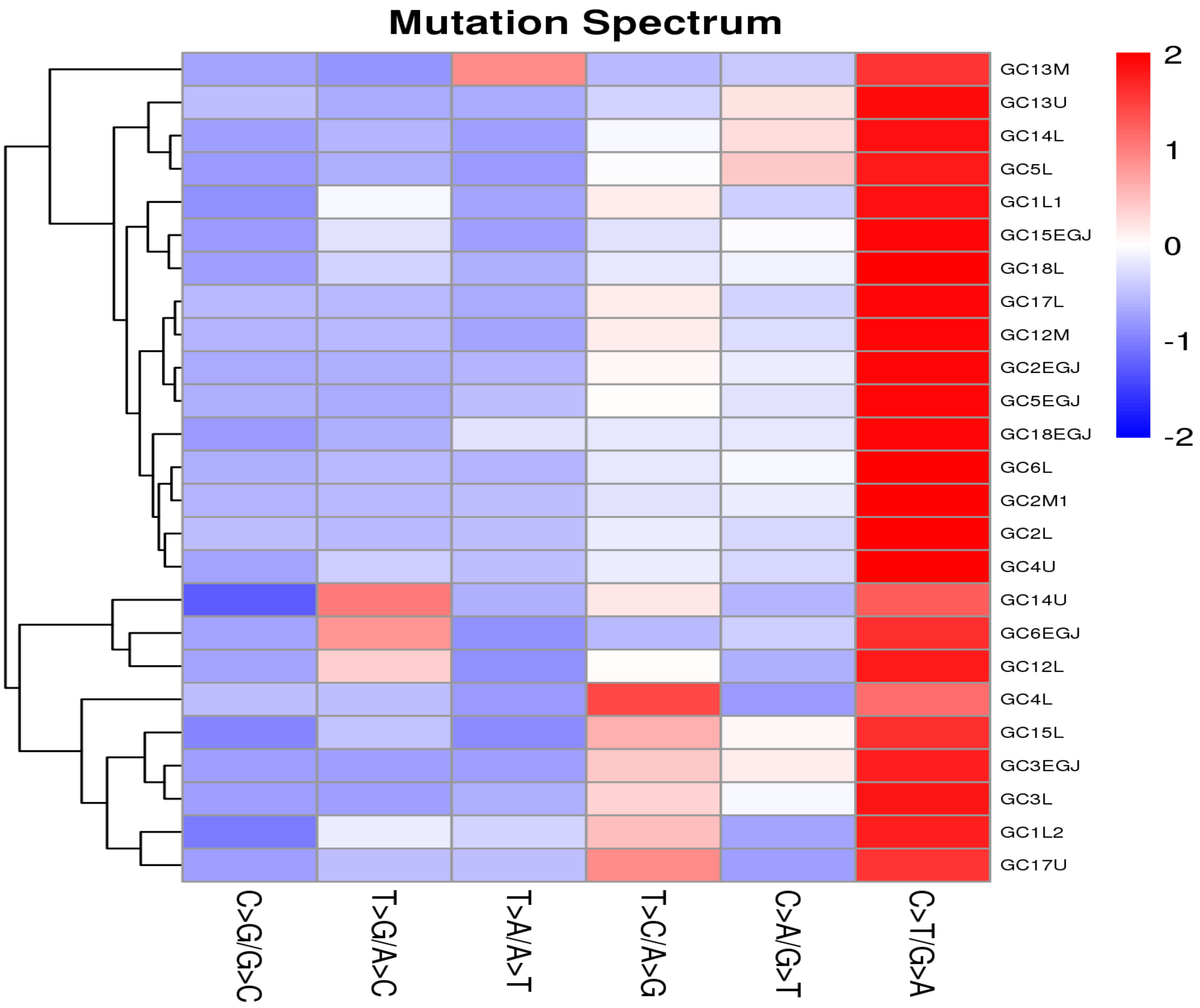

Supplementary Figure 3: Mutation signatures of genetic MGCs

Legend: The chart shows three mutation signatures of all tumor samples. X-axis represents 96 mutation types. Signature A, signature B and signature C indicate three different mutation signatures.

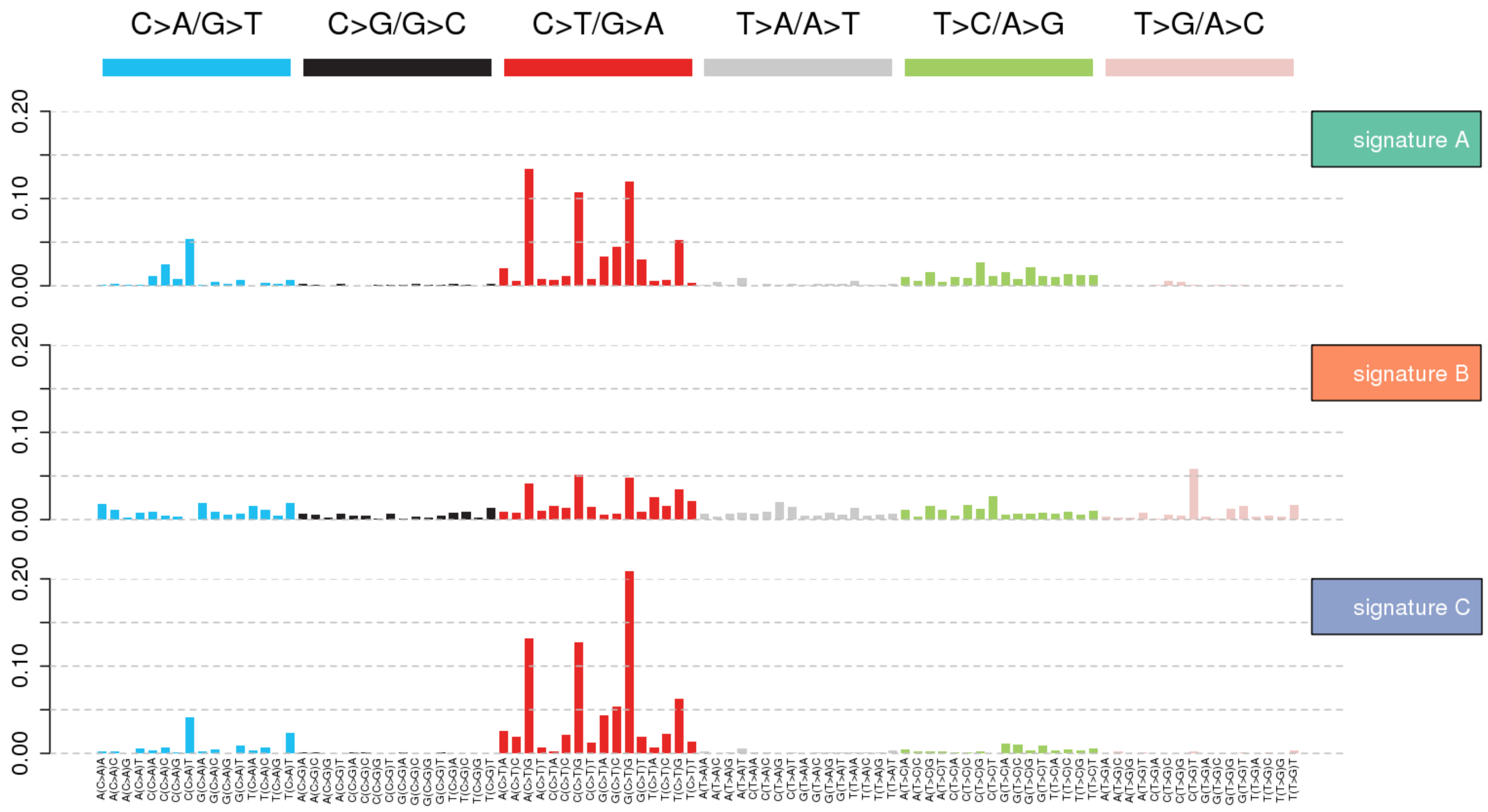

Supplementary Figure 4: Cosine similarity heatplot of identified signatures and known signatures

Legend: The plot shows the proportion of three mutation signatures. The X-axis represents different genetic MGC tumor samples. The Y-axis indicates the relative weight of signatures.

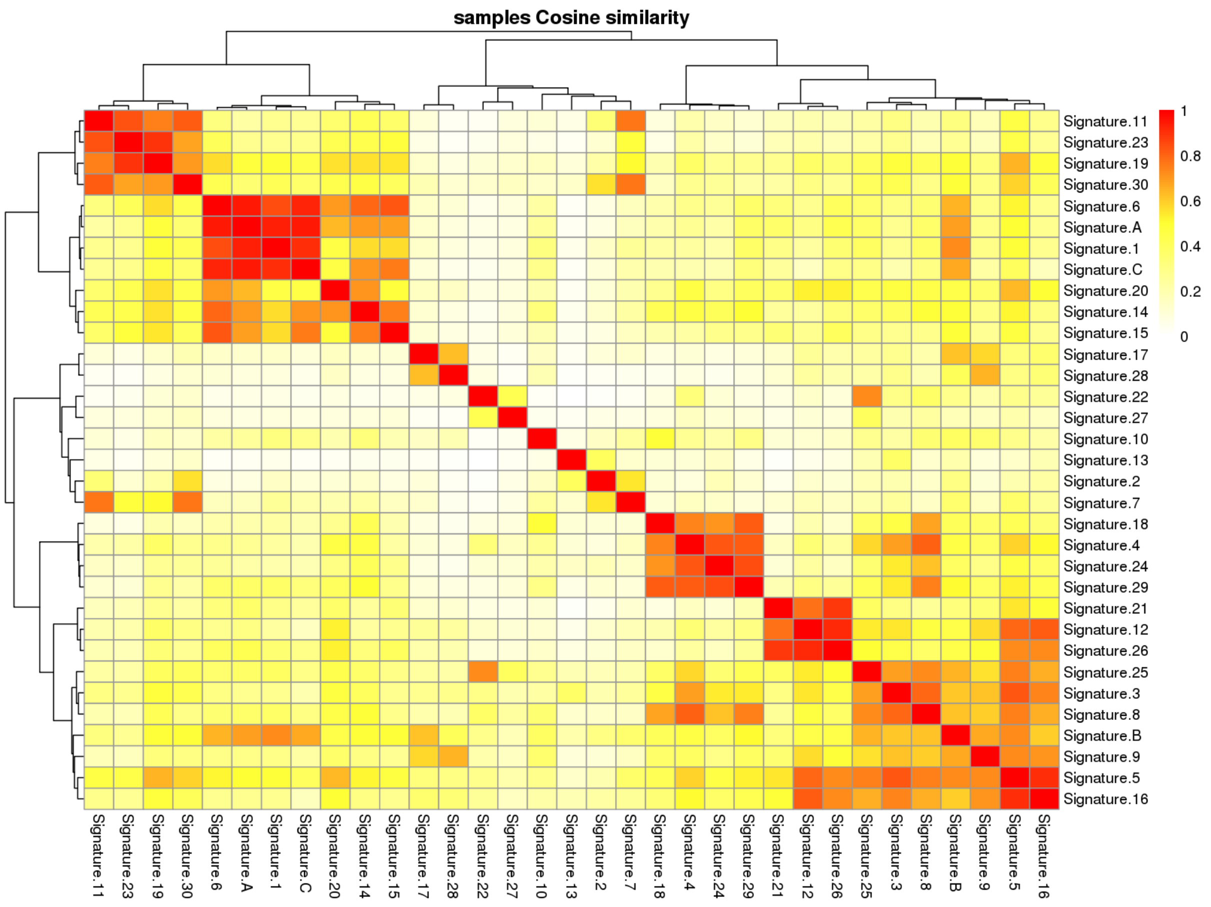

Supplementary Figure 5: The distribution of mutation signatures in genetic MGCs

Legend: The chart shows three mutation signatures of all tumor samples. X-axis represents 96 mutation types. Signature A, signature B and signature C indicate three different mutation signatures.

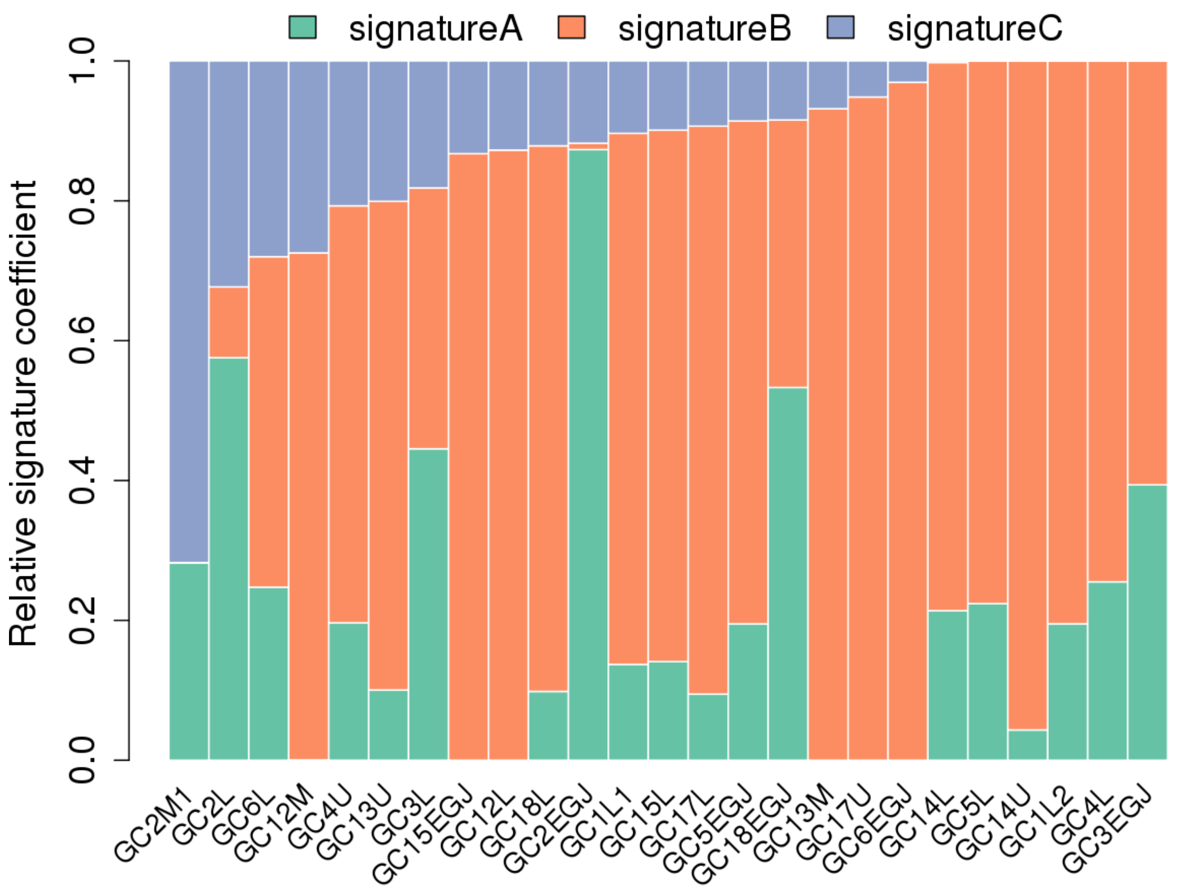

Supplementary Figure 6: The mutational information of driver genes among genetic MGCs

Legend: The bar chart with different colors indicates different protein functional domains. The number below chart represents the length of functional domain. The line means the specific site of mutation.

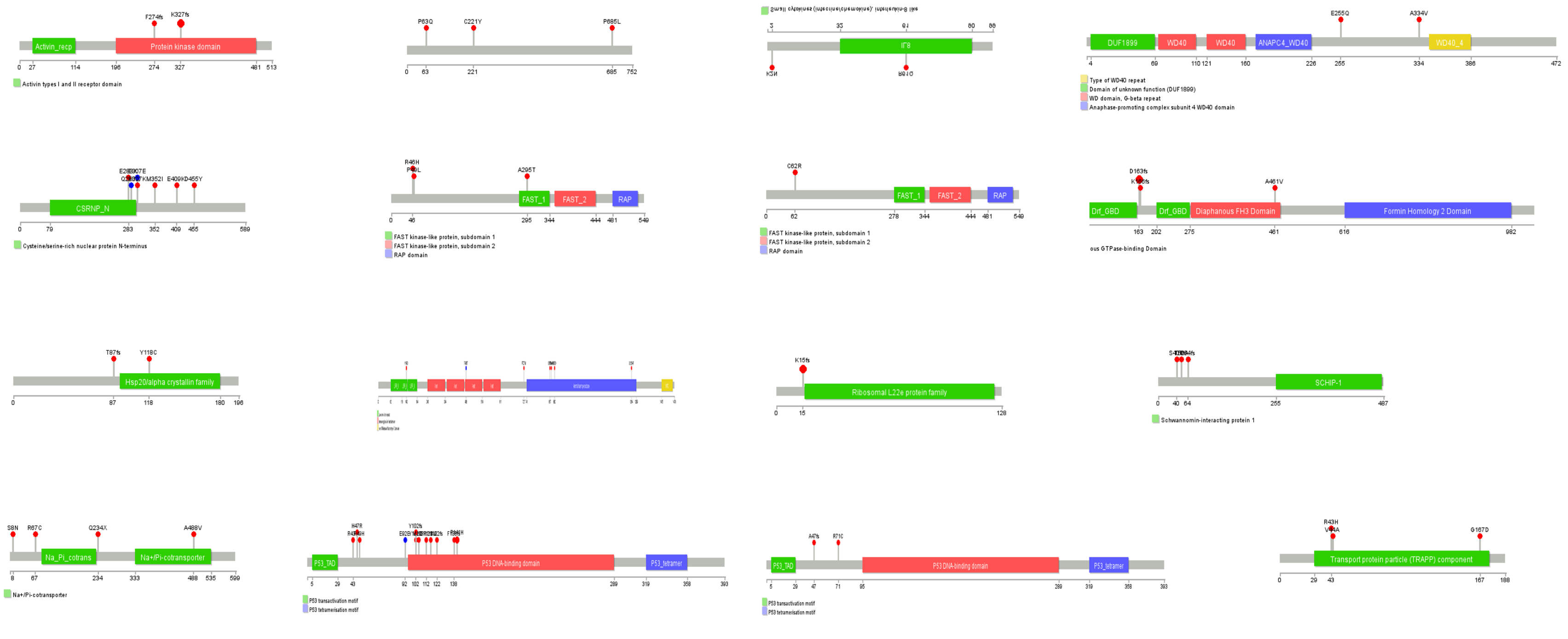

Supplementary Figure 7: The mutational information of SMGs among genetic MGCs

Legend: The bar chart with different color indicates different protein functional domain. The number below chart represents the length of functional domain. The line means the specific site of mutation.

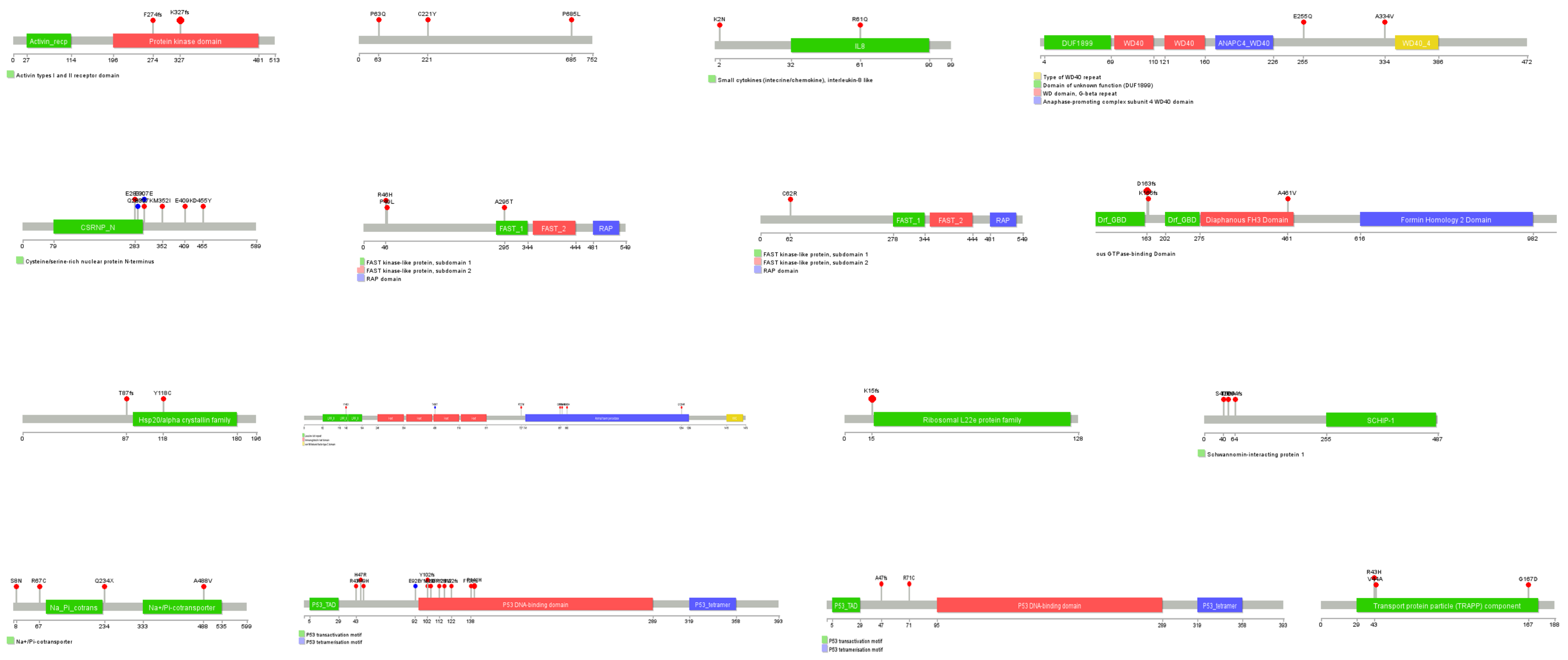

Supplementary Figure 8: Profile of somatic copy number variation on exome data of genetic MGCs

Legend: The chart depicts the distribution of somatic copy number variation (CNV). Red is for CNV gain, green for normal CNV and blue for CNV loss.

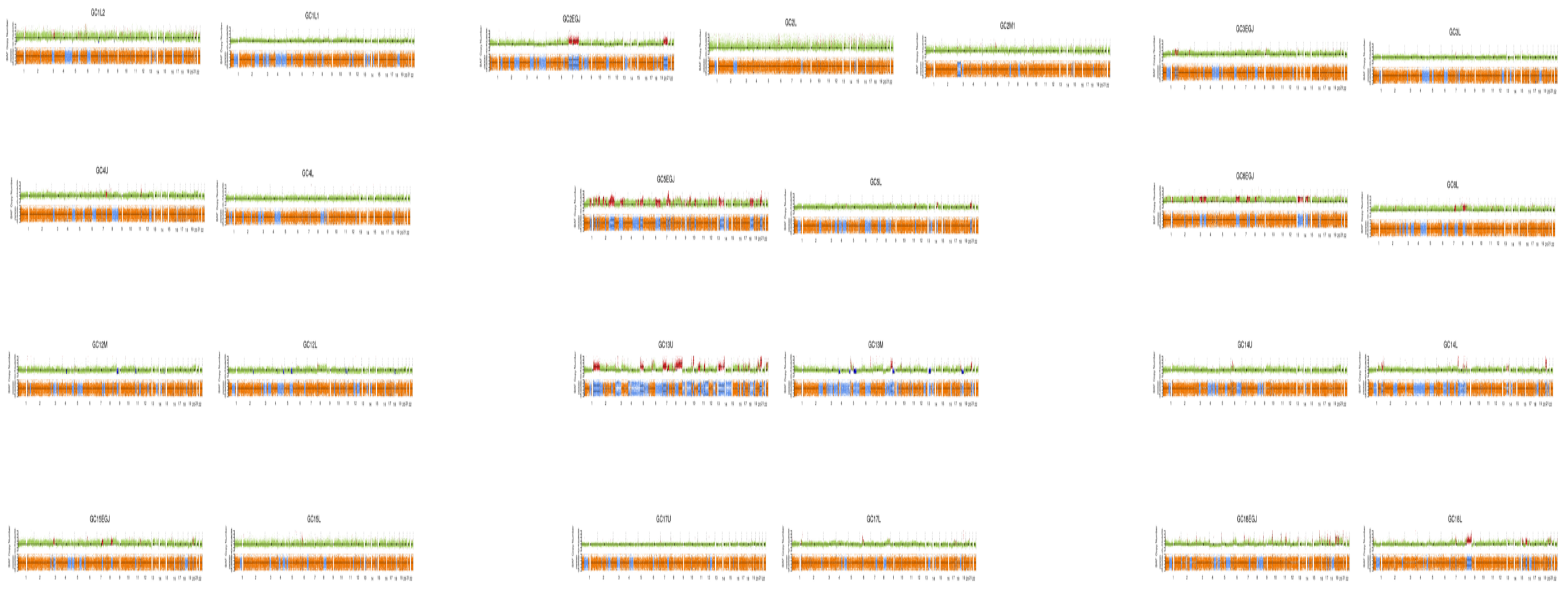

Supplementary Table 1: Information on whole-exome sequencing of 49 samples in 16 MGCs patients

**Sample**	Average_sequencing_depth_on_target	Coverage_of_target_region	Fraction_of_target_covered_with_at_least_10x	Fraction_of_target_covered_with_at_least_50x	Fraction_of_target_covered_with_at_least_100x
**N1**	142	100%	100%	94%	65%
**GC1L1**	266	100%	100%	98%	91%
**GC1L2**	260	100%	100%	97%	89%
**N2**	121	100%	99%	91%	56%
**GC2EGJ**	263	100%	100%	98%	90%
**GC2L**	273	100%	99%	97%	89%
**GC2M1**	256	100%	99%	98%	90%
**N3**	173	100%	100%	93%	66%
**GC3EGJ**	259	100%	100%	98%	90%
**GC3L**	286	100%	100%	98%	89%
**N4**	146	100%	99%	88%	56%
**GC4L**	270	100%	100%	97%	86%
**GC4U**	261	100%	100%	97%	89%
**N5**	133	100%	100%	84%	48%
**GC5EGJ**	272	100%	100%	98%	92%
**GC5L**	286	100%	100%	98%	90%
**N6**	198	100%	100%	94%	70%
**GC6EGJ**	260	100%	100%	98%	90%
**GC6L**	249	100%	100%	98%	89%
**N7**	124	100%	100%	91%	55%
**GC7L**	254	100%	100%	98%	88%
**GC7M**	255	100%	100%	98%	86%
**N8**	137	100%	100%	92%	60%
**GC8L2**	284	100%	100%	98%	90%
**GC8L1**	272	100%	100%	98%	89%
**N9**	152	100%	100%	94%	68%
**GC9L2**	262	100%	100%	98%	89%
**GC9L1**	279	100%	100%	98%	90%
**N12**	137	100%	100%	93%	62%
**GC12L**	254	100%	100%	98%	86%
**GC12M**	263	100%	100%	98%	86%
**N13**	141	100%	100%	92%	62%
**GC13M**	263	100%	100%	98%	86%
**GC13U**	269	100%	100%	97%	85%
**N14**	148	100%	100%	93%	66%
**GC14L**	269	100%	100%	98%	91%
**GC14U**	265	100%	100%	98%	90%
**N15**	129	100%	100%	90%	57%
**GC15EGJ**	261	100%	100%	98%	91%
**GC15L**	264	100%	100%	98%	89%
**N16**	219	100%	100%	97%	83%
**GC16L**	259	100%	100%	98%	90%

**GC16U**	269	100%	100%	98%	88%
**N17**	121	100%	99%	87%	50%
**GC17L**	258	100%	99%	97%	90%
**GC17U**	267	100%	100%	98%	91%
**N18**	131	100%	99%	89%	56%
**GC18EGJ**	286	100%	100%	98%	89%
**GC18L**	261	100%	100%	98%	90%
